# Supplementary material for: Evidence from EXAFS for Different Ta/Ti Site Occupancy in High Critical Current Density Nb3Sn Superconductor Wires
Source: Sci Rep. 2018 Mar 19;8:4798. doi: 10.1038/s41598-018-22924-3 (PMC5859093; doi:10.1038/s41598-018-22924-3)
Supplement: Supplementary file 1 — Supplementary Information [file 41598_2018_22924_MOESM1_ESM.pdf]

Supplementary Information

Evidence from EXAFS for Different Ta/Ti Site Occupancy in High Critical Current Density Nb_3Sn Superconductor Wires

Steve M. Heald,[1] Chiara Tarantini,[2*] Peter J. Lee,[2] Michael Brown,[2] ZuHawn Sung,[2] Arup K. Ghosh,[3] David C. Larbalestier[2]

[1] *Advanced Photon Source, Argonne National Lab, Argonne IL 60439 USA*

[2] *National High Magnetic Field Laboratory, Florida State University, Tallahassee, Florida 32310, USA*

[3]*Retired, formerly Magnet Division, Brookhaven National Laboratory, Upton, New York 11973, USA*

The raw $\chi(k)$ data are shown in Figure S1 for the Ta data and in Figure S2 for the Ti data.

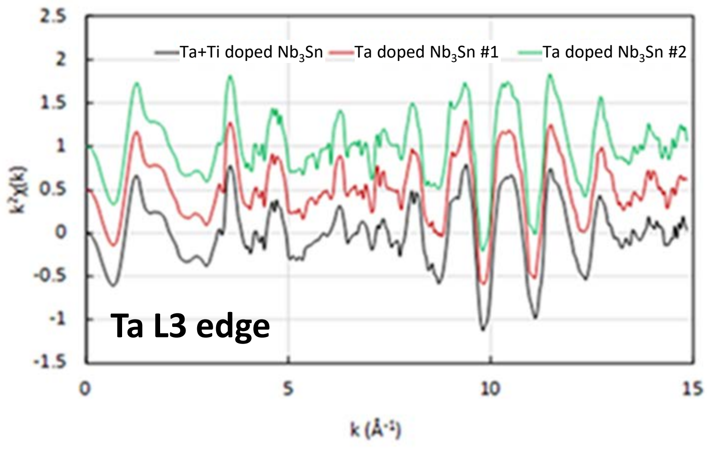

Figure S1. k^2 weighted $\chi(k)$ data for the Ta L3 edge.

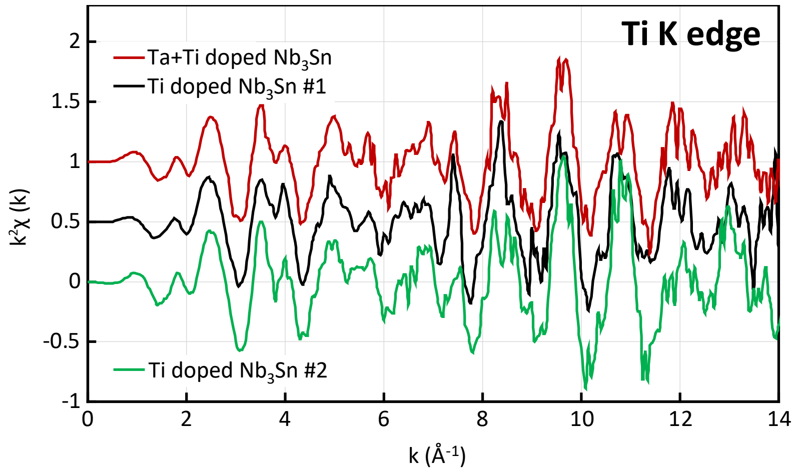

Figure S2. k^2 weighted $\chi(k)$ data for the Ti K edge.
